# A New Class of Heterocycles: 1,4,3,5-Oxathiadiazepane 4,4-dioxides

**DOI:** 10.3390/molecules17021890

**Published:** 2012-02-14

**Authors:** Amel Bendjeddou, Tahar Abbaz, Zine Regainia, Nour-Eddine Aouf

**Affiliations:** 1 Laboratoire de Chimie Organique Appliquée, Groupe de chimie Hétérocyclique, Département de Chimie, Faculté des Sciences, Université d’Annaba, BP 12, 23000, Algérie; 2 Laboratoire de Chimie des Matériaux Organiques, Université de Tébessa, Route de Constantine, Tébessa, 12000, Algérie

**Keywords:** cyclic sulfamides, benzodithiazine dioxides, benzoxathiazepine dioxides

## Abstract

This work reports the synthesis of novel 1,4,3,5-oxathiadiazepanes 4,4-dioxides from the reaction of *N*’-benzyl-*N*-(2-hydroxyethyl)-sarcosine or proline sulfamide with aromatic aldehydes under acid catalysis. To prepare the starting materials *N*-Boc-sulfamide derivatives of sarcosine or proline were alkylated with benzyl alcohol under Mitsunobu reaction conditions, the Boc group was removed chemoselectively by acidolysis, and the resulting product reduced to the corresponding alcohol in good yields.

## 1. Introduction

Sulfamides and their analogs have a rich chemical and biological history and have emerged as a promising class of compounds in drug discovery [1,2]. Sultams (cyclic sulfamides), although not found in Nature, have also shown potent biological activity, including several displaying a wide spectrum of activities [3,4]. The more prominent include a number of benzodithiazine dioxides and benzoxathiazepine 1,1-dioxides displaying anti-HIV-1 activity [5] and the ability to activate glucokinase [6] (type II diabetes), respectively.

In addition, pyrrolo[2,1-*c*]benzodiazepine antibiotics and some of their heterocyclic analogs show anticancer activity [7,8,9,10], it has been proposed that the cytotoxicity and antitumour activity of these compounds results from the formation of a covalent bond between the azomethine unit of the diazepine ring and the C(2)-amino group guanine nucleus in the minor groove of the DNA double spiral [11,12,13,14].

**Figure 1:**
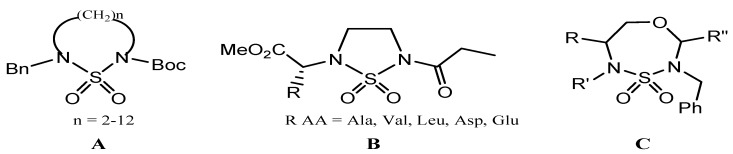
Cyclosulfamide stuctures.

In a continuous of previous work [15,16,17,18,19], we have described a convenient access to a series of n-membered cyclic sulfamides **A** and heterocyclic constrained peptides containing sulfamide groups **B**, starting from natural amino acids, chloroethylamine and chlorosulfonylisocynanate (CSI). In continuation of our efforts to design and synthesize new cyclic sulfamides, we have extended our studies to a series of seven membered heterocyclic compounds **C** containing sulfamide groups (Figure 1), we describe for the first time the first example of a new heterocyclic class 1,4,3,5-oxathiadiazepane 4,4-dioxides which can be described as structural analogs of the anti-HIV-1 compounds mentioned above. This compound is an interesting candidate for pharmaceutical purposes.

## 2. Results and Discussion

**Scheme 1:**
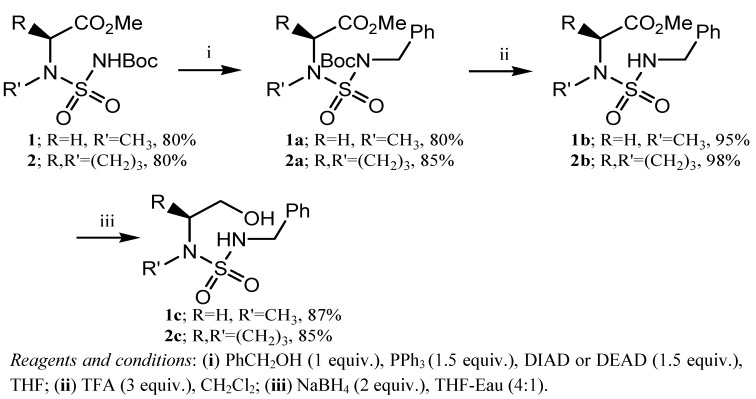
General synthesis of substituted aminoalcohol sulfamides.

In recent years, considerable attention was paid to compounds resulting from the condensation of substituted amino alcohols with aldehydes [20,21,22,23,24]. As outlined in Scheme 1, the substituted amino alcohols (*N*’-benzyl-*N*-(2-hydroxyethyl)-proline or sarcosine sulfamides) **1c** and **2c** were prepared in a three-step reaction sequence starting from (*tert*-butyloxycarbonylsulfonyl) L-amino acid methyl esters **1** and **2**. These compounds were synthesized by sulfamoylation of aminoester derivatives (Pro, Sar) as previously described [18].

In these *tert-*butyloxycarbonylsulfamides **1** and **2**, the Boc (*tert*-butyloxycarbonyl) group increases the acidity of the adjacent NH group and allows an expedient regiospecific alkylation under Mitsunobu conditions [25,26,27,28] using benzylic alcohol, which provides the *N*-substituted Boc-sulfamides **1a** and **2a** in 80% and 85% yield, respectively. Selective cleavage of the *tert*-butyloxycarbonyl protective group with trifluoroacetic acid gives compounds **1b** and **2b** in 95% and 98% yield, respectively. The substituted amino alcohols sulfamides **1c** and **2c** were obtained from the deprotected sulfamides by NaBH_4_ reduction in 87% and 85% yield, respectively.

Substituted 1,4,3,5-oxathiadiazepane 4,4-dioxides were obtained in accordance with the methodology shown in the literature [20]. The substituted amino alcohols **1c** and **2c** were allowed to react with aromatic aldehydes in dichloromethane in a cycolodehydration reaction to obtain the corresponding compounds **1d**–**4d**, **1e**–**4e**. The yields are listed in Table 1. These compounds can furnish after debenzylation new ring opened products by nucleophilic attack by organometallic reagents [27,28].

**Table 1:** General synthesis of substituted oxathiadiazepane 4,4-dioxides. 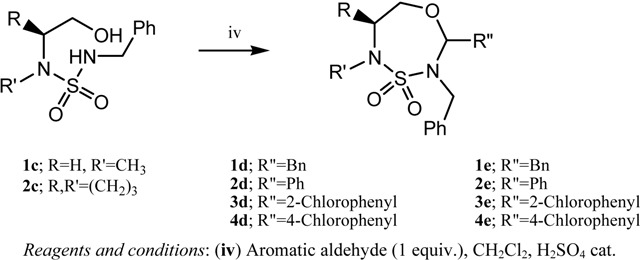

Compounds	Yield (%)
**1d**	50
**2d**	50
**3d**	43
**4d**	41
**1e**	55
**2e**	45
**3e**	40
**4e**	42

In the ^1^H-NMR (CDCl_3_) spectra of proline derivatives **1e**–**4e**, the asymmetric carbon protons of compounds **3e** and **4e** resonate around δ 6.52–6.35 ppm as a singlet peak. Compounds **1e** and **2e** exhibit a doublet of doublets at δ ~ 5.48 ppm and 7.90 ppm, with coupling constants of 3.43, 3.49 Hz and 1.53, 1.57 Hz, respectively. Thus, the asymmetric carbon proton of sarcosine derivatives, showed for compounds **3d** and **4d** a doublet at δ ~ 7.90 ppm and δ ~ 7.50 ppm with coupling constants of 1.47 Hz and 1.49 Hz, respectively, while compound **2d** exhibit a doublet of doublets at δ ~ 7.80 ppm with coupling constants of 1.37, 1.55 Hz, and compound **1d** presents a triplet at δ ~ 5.10 ppm.

## 3. Experimental

### 3.1. General

All commercial chemicals and solvents were used as received. Melting points were determined in open tubes on a Büchi apparatus and are uncorrected. IR spectra were recorded on a Perkin-Elmer Spectrum 1000 spectrophotometer. Microanalyses were performed in the Microanalysis Laboratory of ENSCM (Montpellier). ^1^H and ^13^C-Nuclear Magnetic Resonance spectra were determined on a Brüker AC 250 spectrometer. Chemical shifts are recorded in ppm (δ) and coupling constants (*J*) in Hertz, relative to tetramethylsilane used as internal standard. Multiplicity is indicated as s (singlet), d (doublet), q (quadruplet), m (multiplet) and combinations of these signals. Fast-atom bombardment mass spectra (FAB) were recorded in positive or negative mode with glycerol (G), thioglycerol (GT), or 3-nitrobenzylalcohol (NOBA) as matrix. Optical rotations for solutions in CHCl_3_ were measured with a POLAX model 2L digital polarimeter. All reactions were monitored by Thin Layer Chromatography (TLC) on silica gel Merck 60 F_254_ precoated aluminium plates, developed by spraying with ninhydrin solution. Column chromatography was performed using silica gel 60 (203–400 mesh).

### 3.2. General Synthetic Procedure for Carbamoylation-Sulfamoylation: Preparation of ***1*** and ***2***

A solution of *N*-chlorosulfonyl *tert*-butylcarbamate (0.05 mol) was prepared by addition of *tert*-butanol (408 mL in 50 mL of dichloromethane) to a solution of CSI (7.1 g in the same solvent). The resulting Boc-sulfamoyl chloride solution (25 mL) and triethylamine (17.40 g, 17.1 mL, 0.085 mol) in dichloromethane (100 mL) was added into a suspension of aminoester (0.05 mol) in the same solvent (120 mL) at 0 °C. The reaction was complete in 45 minutes. The reaction mixture was then diluted with dichloromethane (100 mL) and washed with two portions of 0.1 N HCl solution. The organic layer was dried with (Na_2_SO_4_) and concentrated *in vacuo* to give the crude product, which was purified by column chromatography eluting with dichloromethane to give compounds **1** and **2**.

*(S)-Methyl [N-methyl(N’-tert-butyloxycarbonyl)-sulfamoyl]-glycinate* (**1**). Yield = 80%; TLC: Rf = 0.76 (CH_2_Cl_2_-MeOH 9:1); cristallizable oil; IR (KBr) ν cm^−1^: 3200 (NH), 1770, 1763 (C=O), 1360 and 1150 (SO_2_); ^1^H-NMR (CDCl_3_) δ ppm: 7.62 (s, 1H, NH), 4.14 (s, 2H, CH_2_), 3.75 (s, 3H, OCH_3_), 3.04 (s, 3H, NCH_3_), 1.49 (s, 9H, tBu); M.S: (NOBA, FAB > 0): 283 [M+H]^+^, 565. M = 282; Anal. Calcd for C_9_H_18_N_2_O_6_S: C, 38.29; H, 6.38; N, 9.93; S, 11.34; found: C, 38.24; H, 6.43; N, 9.87; S, 11.28.

*(S)-Methyl [N-(N’-tert-butyloxycarbonyl)-sulfamoyl]-prolinate* (**2**). Yield = 80%; TLC: Rf = 0.58 (CH_2_Cl_2_-MeOH 95:5); m.p. = 132–133 °C; [α]_D_ = −9.5 (c = 1; MeOH); IR (KBr) ν cm^−1^: 1730, 1712 (C=O), 1340 and 1150 (SO_2_); ^1^H-NMR (CDCl_3_) δ ppm: 7.52 (s, 1H, NH); M.S: (NOBA, FAB > 0): 309 [M+H]^+^, 208 ([M+H]^+^-Boc). M = 308; Anal. Calcd for C_11_H_20_N_2_O_6_S: C, 42.85; H, 6.69; N, 9.09; S, 10.38; found: C, 42.27; H, 6.53; N, 9.06; S, 10.41.

### 3.3. General Procedure for the Synthesis of *N*-Boc, *N′*-(benzyl)sulfamides ***1a*** and ***2a***

A solution of *N*-alkyl carboxylsulfamide (0.0065 mol, 2 g), triphenylphosphine (2.56 g) and benzylic alcohol (0.7 g) in THF (15 mL) was added dropwise (20 min, 5 °C) to a solution of equimolar quantities of diethyl(diisopropyl)azodicarboxylate (1.97 g) in THF (5 mL), the reaction medium was stirred under an atmosphere of dry nitrogen for about 45 min. TLC reveals the formation of substituted compound (UV, ninhydrin) less polar than its precursor. Oxidoreduction compounds were removed by filtration after precipitation into diethylether. The filtrate was concentred and the crude residue was purified by column chromatography eluted with dichloromethane. 

*(*S*)-Methyl [*N*-(*N′*-tert-butyloxycarbonyl,* N′-*benzyl)-sulfamoyl]-glycinate* (**1a**). Yield = 80%; TLC: Rf = 0.76 (CH_2_Cl_2_-MeOH 95:5); m.p. = 72 °C; ^1^H-NMR (CDCl_3_) δ ppm: 7.34 (m, 5H, ArH), 4.86 (s, 2H, CH_2_-ph), 4.04 (s, 2H, CH_2_-N), 3.72 (s, 3H, OCH_3_), 2.84 (s, 3H, CH_3_), 1.49 (s, 9H, tBu); M.S: (NOBA, FAB > 0): 373 [M+H]^+^. M = 372; Anal. Calcd for C_16_H_24_N_2_O_6_S: C, 51.61; H, 6.45; N, 7.52; S, 8.60; found: C, 51.43; H, 6.38; N, 7.49; S, 8.62.

*(*S*)-Methyl [*N*-(*N′*-tert-butyloxycarbonyl,* N′-*benzyl)-sulfamoyl]-prolinate* (**2a**). Yield = 85%; TLC: Rf = 0.73 (CH_2_Cl_2_-MeOH 95:5); m.p. = 73 °C; ^1^H-NMR (CDCl_3_) δ ppm: 7.36 (m, 5H, ArH), 4.86 (m, 1H, CH), 4.66 (2dd, 2H, *J* = 3.14, 5.45 Hz, CH_2_-Ph), 3.72 (s, 3H, OCH_3_), 3.48 (m, 1H, CH*), 3.15 (m, 1H, CH), 1.88–2.08 (m, 4H, 2CH_2_), 1.48 (s, 9H, tBu); M.S: (NOBA, FAB > 0): 399 [M+H]^+^. M = 398; Anal. Calcd for C_18_H_26_N_2_O_6_S: C, 54.71; H, 6.53; N, 7.038; S,8.04; found: C, 54.65; H, 6.62; N, 7.05; S, 8.13.

### 3.4. General Deprotection Procedure: Preparation of ***1b*** and ***2b***

A solution of trifluoroacetic acid (50% in dichloromethane; 3 equiv.) was dropwise added into a stirred solution of substituted *N*-carboxylsulfamide(0.92 g, 2.6 mmol) in dichloromethane (10 mL) at 0 °C. The reaction midium was stirred during two hours, concentrated under reduced pressure and coevaporated with diethylether. The residue was purified by column chromatography eluted with dichloromethane (or recrystallized from an AcOEt-hexane mixture) to afford deprotected sulfamides **1b** and **2b**.

N*[*N′*-Benzyl]sulfamoylglcinate de methyl* (**1b**). Yield = 95%; TLC: Rf = 0.55 (CH_2_Cl_2_-MeOH 95:5); m.p. = 100–102 °C; IR (KBr) ν cm^−1^: 3312 (NH), 1360 and 1160 (SO_2_); ^1^H-NMR (CDCl_3_) δ ppm: 7.34 (m, 5H, ArH), 5.70 (t, 1H, NH), 4.30 (d, 2H, *J* = 6.0 Hz, CH_2_-Ph), 4.0 (s, 2H, CH_2_), 3.75 (s, 3H, OCH_3_), 2.90 (s, 3H, CH_3_); M.S: (NOBA, FAB > 0): 273 [M+H]^+^, 546; M = 272; Anal. Calcd for C_11_H_16_N_2_O_4_S: C, 48.52; H ,5.88; N ,10.29; S ,11.76; found: C, 48.39; H, 5.80; N, 10.25; S, 11.74.

N*[*N′*-Benzyl]sulfamoylprolinate de methyl* (**2b**). Yield = 98%; TLC: Rf = 0.60 (CH_2_Cl_2_-MeOH 95:5); oil; IR (KBr) ν cm^−1^: 3320 (NH), 1355 and 1159 (SO_2_); ^1^H-NMR (CDCl_3_) δ ppm: 7.34 (m, 5H, ArH), 5.96 (m, 1H, NH), 4.41 (dd, 1H, J = 3.81, 4.70Hz, C*H), 4.30 (2dd, 2H, *J* = 3.11, 4.70 Hz, CH_2_-Ph), 3.70 (s, 3H, OCH_3_), 3.46 (m, 2H, CH_2_N), 2.20–2.0 (m, 4H, CH_2_β and CH_2_γ); M.S: (NOBA, FAB > 0): 299 [M+H]^+^. M = 298; Anal. Calcd for C_13_H_18_N_2_O_4_S: C, 52.34; H, 6.04; N, 9.39; S, 10.73; found: C, 52.29; H, 6.00; N, 9.37; S, 10.68.

### 3.5. General Reduction Procedure; Preparation of ***1c*** and ***2c***

Deprotected product **1b** or **2b** (3.30 mmol) in THF (15 mL) was added dropwise to a suspension of NaBH_4_ (0.006 mol) in THF-Water (4:1, v/v, 20 mL) at 0 °C. When the addition was complete, the reaction mixture was acidified slowly with HCl 5% and concentrated *in vacuo*. The aqueous layer was extracted with ethyl acetate (3 × 150 mL). The combined organic layers were dried (Na_2_SO_4_) and concentrated *in vacuo*. The crude product was purified by column chromatography eluted with dichlomethane-methanol (90:10).

N*-Methyl(*N′*-benzylsulfamoyl) glycinol* (**1c**). Yield = 87%; TLC: Rf = 0.50 (CH_2_Cl_2_-MeOH 95:5); m.p. = 98 °C; IR (KBr) ν cm^−1^: 3315 (NH), 1330 (OH); ^1^H-NMR (CDCl_3_) δ ppm: 7.35 (m, 5H, ArH), 4.72 (t, 1H, NH), 4.39 (d, 2H, *J* = 5.99 Hz, CH_2_-Ph), 3.78 (t, 2H, CH_2_OH), 3.35 (t, 2H, CH_2_N), 2.88 (s, 3H, CH_3_), 1.98 (s, 1H, OH); M.S: (ESI+): 267 [M+Na]^+^, 511 [2M+Na]^+^; M = 244; Anal. Calcd for C_10_H_16_N_2_O_3_S: C, 49.18; H, 6.55; N, 11.47; S, 13.11; found: C, 49.14; H, 6.52; N, 11.45; S, 13.20.

N*-Methyl(*N′*-benzylsulfamoyl) prolinolnol* (**2c**). Yield = 85%; TLC: Rf = 0.48 (CH_2_Cl_2_-MeOH 95:5); m.p. = 109–112 °C; IR (KBr) ν cm^−1^: 3320 (NH), 1370 (OH); ^1^H-NMR (CDCl_3_) δ ppm: 7.30 (m, 5H, ArH), 5.0 (t, 1H, NH), 4.25 (d, 2H, *J* = 5.5.75 Hz, CH_2_-Ph), 3.81 (m, 1H, CH*), 3.55 (m, 2H, CH_2_OH), 3.30 (t, 2H, CH_2_N), 2.31 (band large, s, 1H, OH), 1.85 (m, 4H, CH_2_β and CH_2_γ); M.S: (ESI+): 293 [M+Na]^+^, 563 [2M+Na]^+^; M = 270; Anal. Calcd for C_12_H_18_N_2_O_3_S: C, 53.33; H, 6.66; N, 10.37; S, 11.85; found: C, 53.31; H, 6.60; N, 10.39; S, 11.89.

### 3.6. General Procedure for the Preparation of 1,4,3,5-Oxathiadiazepanes 4,4-dioxides ***1d**–**4d**, **1e**–**4e***

Compounds **1c** and **2c** (0.01 mol) were dissolved separately in dichloromethane (25 mL), and the aromatic aldehyde (0.01 mol) was added. A drop of concentrated sulfuric acid was also added, and the reaction mixture was stirred for 3h at room temperature. The reaction mixture was washed with a 5% solution of sodium bicarbonate, water and then with brine. The organic layer was dried over anhydrous sodium sulfate, and evaporated under reduced pressure on a rotary evaporator. The residue was purified by column chromatography eluting with dichloromethane to give the 1,4,3,5-oxathiadiazepanes 4,4-dioxide.

*(*N^3^*,2)-Dibenzyl, *N^5^*-methyl 1,4,3,5-oxathidiazepane 4,4-dioxyde* (**1d**). Yield = 59%; TLC: Rf = 0.75 (CH_2_Cl_2_); m.p. = 82–85 °C; IR (KBr); ^1^H-NMR (CDCl_3_) δ ppm: 7.30 (m, 10H, 2ArH), 7.09 (m, 2H, C*-CH_2_-ph), 5.1 (t, *J* = 5.37, 1H, CH*), 4.55 (s, 2H, CH_2_-N), 3.90 (m, 2H, CH_2_-O), 2.97 (S, 3H, CH_3_-N), 2.85 (m, 2H, CH_2_-N); ^13^C-NMR (CDCl_3_) δ ppm: 37.80, 41.34, 49.74, 52.80, 68, 89.31, 128; M.S: (ESI+): 369 [M+Na]^+^; M = 346; Anal. Calcd for C_18_H_21_N_2_O_3_S: C, 62.42; H, 6.06; N, 8.09; S, 9.24; found: C, 62.44; H, 6.02; N, 8.03; S, 9.20.

N^3^*-Benzyl, *N^5^*-methyl, 2-phenyl 1,4,3,5-oxathidiazepane 4,4-dioxyde* (**2d**). Yield = 50%; TLC: Rf = 0.75 (CH_2_Cl_2_); oil; ^1^H-NMR (CDCl_3_) δ ppm: 7.80 (dd, *J* = 1.37, 1.55 Hz, 1H, CH*), 7.30 (m, 10H, 2Ar), 4.35 (t, 2H, CH_2_-O), 4.25 (dd, *J* = 27.57, 27.52 Hz, 2H, CH_2_-Ph), 3.30 (t, 2H, CH_2_-N), 2.90 (S, 3H, CH_3_-N); ^13^C-NMR (CDCl_3_) δ ppm: 37.80, 49.70, 52.80, 68, 87.50, 128.50; (ESI+): 355 [M+Na]^+^; M = 332; Anal. Calcd for C_17_H_20_N_2_O_3_S: C, 61.44; H, 6.02; N, 8.43; S ,9.63; found: C, 61.40; H, 6.10; N, 8.50; S, 9.67.

N^3^*-Benzyl, *N^5^*-methyl, 2-(2-chlorophenyl) 1,4,3,5-oxathidiazepane 4,4-dioxyde* (**3d**). Yield = 43%; TLC: Rf = 0.80 (CH_2_Cl_2_); m.p. = 80–82 °C; ^1^H-NMR (CDCl_3_) δ ppm: 7.90 (d, *J* = 1.47 Hz, 1H, CH*), 7.40 (m, 9H, 2Ar), 4.50 (t, *J* = 5.49 Hz, 2H, CH_2_-O), 4.20 (d, *J* = 5.98 Hz, 2H, CH_2_-Ph), 3.57 (t, *J* = 5.49 Hz , 2H, CH_2_-N), 2.92 (S, 3H, CH_3_-N); ^13^C-NMR (CDCl_3_) δ ppm: 37.80, 49.50, 53, 68.4, 84.60, 129, 132; M.S: (ESI+): 389 [M+Na]^+^; M = 366; Anal. Calcd for C_17_H_19_N_2_O_3_SCl: C,55.73; H, 5.19; N, 7.65; S, 8.74; found: C, 55.68; H, 5.14; N, 7.59; S, 8.70.

N^3^*-Benzyl, *N^5^*-methyl, 2-(4-chlorophenyl) 1,4,3,5-oxathidiazepane 4,4-dioxyde* (**4d**). Yield = 41%; TLC: Rf = 0.80 (CH_2_Cl_2_); m.p. = 80–82 °C; ^1^H-NMR (CDCl_3_) δ ppm: 7.50 (d, *J* = 1.49 Hz, 1H, CH*), 7.20 (m, 9H, 2Ar), 4.30 (t, *J* = 1.63 Hz, 2H, CH_2_-O), 4.1 and 4.4 (2d, *J* = 15.89 Hz, 1H and *J* = 15.91 Hz, 1H, CH_2_-Ph), 3.5 (t, *J* = 1.49, 2H, CH_2_-N), 3.10 (s, 3H , CH_3_-N); ^13^C-NMR (CDCl_3_) δ ppm: 37.80, 49.50, 55.90, 68.3, 85, 129, 131.5; M.S: (ESI+): 389 [M+Na]^+^; M = 366; Anal. Calcd for C_17_H_19_N_2_O_3_SCl: C, 55.73; H, 5.19; N, 7.65; S, 8.74; found: C, 55.76; H, 5.23; N, 7.72; S, 8.70.

*(*N^3^*,2)-Dibenzyl, (*N^5^*,6)-trimethylene 1,4,3,5-oxathiadiazepane 4,4-dioxide* (**1e**). Yield = 55%; TLC: Rf = 0.82 (CH_2_Cl_2_); m.p. = 107–108 °C; ^1^H-NMR (CDCl_3_) δ ppm: 7.30 (m, 10H, 2ArH), 5.48 (dd, *J* = 3.43, *J* = 3.49 Hz, 1H, C_1_H*), 5.31 (s, 2H, CH_2_-N), 4.81 and 4.55 (2d, *J* = 16.77 Hz, 1H and *J* = 16.68 Hz, 1H, CH_2_-Ph), 3.81 (m, 2H, CH_2_-O), 3.50 (m, 3H, CH_2_-N and C_2_H*-N), 1.35 (m, 4H, CH_2_β and CH_2_γ); ^13^C-NMR (CDCl_3_) δ ppm: 24.80, 28.97, 29.71, 41.41, 48.19, 59.15, 73.06, 88.98, 129; M.S: (ESI+): 395 [M+Na]^+^; M = 372; Anal. Calcd for C_20_H_24_N_2_O_3_S: C, 64.5; H, 6.45; N, 7.52; S, 8.60; found: C, 64.49; H, 6.46; N, 7.47; S, 8.65.

*2-Phenyl, *N^3^*-benzyl, (*N^5^*,6)-trimethylene 1,4,3,5-oxathiadiazepane 4,4-dioxide* (**2e**). Yield = 45%; TLC: Rf = 0.79 (CH_2_Cl_2_); m.p. = 97–99 °C; ^1^H-NMR (CDCl_3_) δ ppm: 7.90 (dd, *J* = 1.53, *J* = 1.57 Hz, 1H, C_1_H*), 7.45 (m, 10H, 2ArH), 4.30 (s, 2H, CH_2_-Ph), 3.90 (m, 1H, C_2_H*), 3.70–3.60 (m, 2H, CH_2_-O), 3.50 (m, 2H, CH_2_-N), 1.80 (m, 4H, CH_2_β and CH_2_γ); ^13^C-NMR (CDCl_3_) δ ppm: 24.80, 28.97, 41, 48.20, 59.15, 72.80, 89.80, 129; M.S: (ESI+): 381 [M+Na]^+^; M = 358; Anal. Calcd for C_19_H_22_N_2_O_3_S: C, 63.68; H, 6.14; N, 7.82; S, 8.93; found: C, 63.72; H, 6.16; N, 7.79; S, 8.89.

*2-(2-Chlorophenyl), *N^3^*-benzyl, (*N^5^*,6)-trimethylene 1,4,3,5-oxathiadiazepane 4,4-dioxide* (**3e**). Yield = 40%; TLC: Rf = 0.80 (CH_2_Cl_2_); oil; ^1^H-NMR (CDCl_3_) δ ppm: 6.94 (m, 9H, 2ArH), 6.52 (s, 1H, C_1_H*), 4.50 (dd, *J* = 15.81, *J* = 16.24 Hz, 2H, CH_2_-Ph), 4.10–3.90 (m, 3H, CH_2_-O and C_2_H*), 3.60 (t, 2H, CH_2_-N), 2.08 (m, 2H, CH_2_β,), 1.60 (m, 2H, CH_2_γ); ^13^C-NMR (CDCl_3_) δ ppm: 24.80, 28.97, 41, 48.20, 59.15, 72.80, 89.80, 129, 131.5; M.S: (ESI+): 415 [M+Na]^+^; M = 392.5; Anal. Calcd for C_19_H_21_N_2_O_3_SCl: C, 58.08; H, 5.35; N, 7.13; S, 8.15; found: C, 58.00; H, 5.29; N, 7.09; S, 8.20.

*2-(4-Chlorophenyl), *N^3^*-benzyl, (*N^5^*,6)-trimethylene 1,4,3,5-oxathiadiazepane 4,4-dioxide* (**4e**). Yield = 42%; TLC: Rf = 0.78 (CH_2_Cl_2_); yellow powder, m.p. = 154–157 °C; ^1^H-NMR (CDCl_3_) δ ppm: 7.25 (m, 9H, 2ArH), 6.35 (s, 1H, C_1_H*), 4.48 (dd, *J* = 15.85, *J* = 16.21 Hz, 2H, CH_2_-Ph), 4.11 (m, 1H, C_2_H*), 3.85 (m, 2H, CH_2_-O), 3.55 (m, 2H, CH_2_-N), 2.15 (m, 4H, CH_2_β and CH_2_γ,); ^13^C-NMR (CDCl_3_) δ ppm: 24.80, 28.97, 41, 48.20, 59.15, 72.80, 89.80, 129, 131; M.S: (ESI+): 415 [M+Na]^+^, 807 [2M+Na]^+^; M = 392.5; Anal. Calcd for C_19_H_21_N_2_O_3_SCl: C, 58.08; H, 5.35; N, 7.13; S, 8.15; found: C, 58.10; H, 5.39; N, 7.16; S, 8.19.

## 4. Conclusions

In conclusion, we have successfully prepared a new class of seven-membered heterocyclic substituted 1,4,3,5-oxathiadiazepanes 4,4-dioxides via a simple strategy. The biological evaluation of the resulting compounds, their stereochemical study, reopening after debenzylation by nucleophilic attack by organometallic reagents and their incorporation into biomolecule analogues are currently underway and will be reported in due course.
